# Ferroptosis inducers enhanced cuproptosis induced by copper ionophores in primary liver cancer

**DOI:** 10.1186/s13046-023-02720-2

**Published:** 2023-06-06

**Authors:** Weikai Wang, Kaizhong Lu, Xin Jiang, Qi Wei, Liyuan Zhu, Xian Wang, Hongchuan Jin, Lifeng Feng

**Affiliations:** 1 Laboratory of Cancer Biology, Key Lab of Biotherapy in Zhejiang Province, Sir Run Run Shaw Hospital, School of Medicine, Cancer Center of Zhejiang University, Zhejiang University, Hangzhou, Zhejiang China; 2 Department of Medical Oncology, Sir Run Run Shaw Hospital, School of Medicine, Zhejiang University, Hangzhou, Zhejiang China

**Keywords:** Cuproptosis, Ferroptosis, Copper ionophores, Lipoylation, Ferredoxin 1 (FDX1)

## Abstract

**Introduction:**

Cuproptosis and ferroptosis are the two newly defined metal-related regulated cell death. However, the crosstalk between cuproptosis and ferroptosis is obscure.

**Materials and methods:**

We analyzed the effect of ferroptosis inducers on copper ionophores-induced cell death through CCK-8 assay. Cuproptosis was studied using immunofluorescence and protein soluble-insoluble fraction isolation. GSH assay, qRT-PCR and western blot were adopted to explore the machinery of ferroptosis inducers enhanced cuproptosis. And mouse xenograft model was built to detect the synergy effect of elesclomol-Cu and sorafenib in vivo.

**Results:**

Herein we found that ferroptosis inducers sorafenib and erastin could enhance cuproptosis in primary liver cancer cells by increasing copper dependent lipoylated protein aggregation. Mechanically, sorafenib and erastin upregulated protein lipoylation via suppressing mitochondrial matrix-related proteases mediated ferredoxin 1 (FDX1) protein degradation, and reduced intracellular copper chelator glutathione (GSH) synthesis through inhibiting cystine importing.

**Discussion/Conclusion:**

Our findings proposed that combination of ferroptosis inducers and copper ionophores to co-targeting ferroptosis and cuproptosis could be a novel therapeutic strategy for primary liver cancer.

**Supplementary Information:**

The online version contains supplementary material available at 10.1186/s13046-023-02720-2.

## Introduction

The primary liver cancer (Hereafter referred as liver cancer) has a high incidence globally, ranking eighth for morbidity and sixth for mortality [1]. Liver cancer mainly includes hepatocellular carcinoma (HCC) (accounting for 75-85%) and intrahepatic cholangiocarcinoma (ICC) (10-15%) [2]. HCC and ICC are both aggressive malignancies showing extremely poor prognosis and lacking effective therapeutics. Therefore, it is of great significance to develop novel therapeutic strategies for liver cancer.

Metal ion metabolism homeostasis is crucial for cell viability maintenance [3]. Recent findings uncovered an urgent demand of metal ions (iron and copper etc.) for cancer cell proliferation and progression, which encouraged cancer cell to remodel metal ion metabolism network to maintain a higher level of intracellular metal ions [4–6]. Therefore, new therapies targeting metal ion metabolism such as inducing iron-dependent ferroptosis have shown a promising prospect in cancer therapy [7–9]. Ferroptosis is a new type of regulated cell death characterized by ferrous ion mediated lipid peroxidation [7, 10, 11]. Ferroptosis inducers (FINs) have been proved to exhibit great potential especially in ferroptosis-vulnerable cancer and overcoming resistance to various therapies such as radiotherapy, chemotherapy and immunotherapy [12–19]. For instance, class I FINs including sorafenib and erastin, which induce ferroptosis via suppressing intracellular glutathione (GSH) synthesis to inhibit glutathione peroxidase 4 (GPX4)-mediated antioxidant reaction, have exhibited great efficacy in preclinical cancer models or clinic administration of liver cancer patients [7, 8]. Cuproptosis is another emerging metal ion-dependent regulated cell death which depends on intracellular copper. Acting as the scaffold, excessive intracellular copper directly binds to lipoylated components in the tricarboxylic acid (TCA) cycle, which causes the aggregation of lipoylation-related proteins, thus disrupting protein homeostasis and finally triggering a distinct modality of cell death [20]. Therefore, copper ionophores such as elesclomol have attracted great interest in cancer therapy. As the two newly identified metal ion-based regulated cell death forms, whether there is an interplay between ferroptosis and cuproptosis needed urgently investigations.

Herein, we identified that ferroptosis inducers sorafenib and erastin intensified copper ionophores-induced cuproptosis in liver cancer through upregulating protein lipoylation and suppressing intracellular GSH synthesis. Furthermore, combination of FINs and cuproptosis-inducing agents significantly inhibited liver cancer growth in vivo via elevated cuproptosis. Therefore, the synergy ferroptosis inducers with copper ionophores might be a novel and more effective anti-liver cancer strategy.

## Materials and methods

### Cells, antibodies and chemicals

Human primary liver cancer cell lines including HCC cells MHCC-97 H, Huh7, and ICC cells QBC939, CCLP1 were all purchased from Cell Bank of the Typical Culture Preservation Committee, Chinese Academy of Sciences (Shanghai, China). MHCC-97 H, Huh7, QBC939 and CCLP1 cells were cultured in the DMEM medium (Invitrogen, Shanghai, China). All mediums were supplemented with 10% FBS and 100U/mL penicillin–streptomycin. The following commercially available antibodies were used for Western blotting: GAPDH (Abcam, ab75834), FDX1 (Abcam, ab108257), Actin (Abclonal, ac026), DLAT (CST, 12,362S), LIAS (Proteintech, 11577-1-AP), Lipoic acid antibody (Abcam, ab58724), AFG3L2 (Proteintech, 14631-1-AP), ATF4 (CST, 11,815), p62 (MBL, PM045). The chemical reagents used in this study included L-Glutathione (reduced) (MCE, HY-D0187), Sorafenib(Selleck, S7397), Erastin(MCE, HY-15,763), Ammonium tetrathiomolybdate (TTM) (Macklin, A828261), Elesclomol (MCE, HY-12,040), Ditiocarb sodium (MCE, HY-B1637), Baf-A1 (Sigma Aldrich, B1793), cycloheximide (Sigma Aldrich, C7698), MG-132 (Sigma Aldrich, 474,790), MitoTracker Red CMXRos (Thermo Fisher Scientific, M7512), Z-VAD-FMK (MCE, HY-16658B), BOC-D-FMK (MCE, HY-13,229), Tetraethylenepentamine (TEPA) (Macklin, T818822), BODIPY™ 581/591 C11 (Invitrogen, D3861).

### siRNAs transfection

siRNAs mentioned in this article were synthesized by GenePharma Company (Shanghai, China), and transfected into cells with Lipofectamine™ RNAiMAX transfection reagent (Thermo Fisher Scientific) at a final concentration of 20–50 nM. All sequences of siRNAs used were listed as follow: siNC sense (5’-3’) UUCUCCGAACGUGUCACGUTT, antisense (5’-3’) ACGUGACACGUUCGGAGAATT, siFDX1 1# sense (5’-3’) GUGAUUCUCUGCUAGAUGUTT, antisense (5’-3’) ACAUCUAGCAGAGAAUCACTT, siFDX1 2# sense (5’-3’) CUAACAGACAGAUCACGGUTT, antisense (5’-3’) ACCGUGAUCUGUCUGUUAGTT, siLIAS 1# sense (5’-3’) GCACCUGGGAUGAAUAUAATT, antisense (5’-3’) UUAUAUUCAUCCCAGGUGCTT, siLIAS 2# sense (5’-3’) GGUGCGUUCUUCAUAUAAATT, antisense (5’-3’) UUUAUAUGAAGAACGCACCTT, siAFG3L2 1# sense (5’-3’) GGACGCUUUACCGAUUUGUTT, antisense (5’-3’) ACAAAUCGGUAAAGCGUCCTT, siAFG3L2 2# sense (5’-3’) GGAAGGACUUUGUCAAUAATT, antisense (5’-3’) UUAUUGACAAAGUCCUUCCTT, siCLPP 1# sense (5’-3’) GCGAGCGCGCCUAUGACAUTT, antisense (5’-3’) AUGUCAUAGGCGCGCUCGCTT, siCLPP 2# sense (5’-3’) CCCGAUCGAUGACAGCGUUTT, antisense (5’-3’) AACGCUGUCAUCGAUCGGGTT, siLONP1 1# sense (5’-3’) GUCGGCGUCUUUCUAAAGATT, antisense (5’-3’) UCUUUAGAAAGACGCCGACTT, siLONP1 2# sense (5’-3’) GGGACAUCAUUGCCUUGAATT, antisense (5’-3’) UUCAAGGCAAUGAUGUCCCTT, siBAX 1# sense (5’-3’), CGGAACUGAUCAGAACCAUTT, antisense (5’-3’), AUGGUUCUGAUCAGUUCCGTT, siBAX 2# sense (5’-3’), CAUCAGAUGUGGUCUAUAATT, antisense (5’-3’), UUAUAGACCACAUCUGAUGTT, siBAK1 1# sense (5’-3’), ACAUCAACCGACGCUAUGATT, antisense (5’-3’), UCAUAGCGUCGGUUGAUGUTT, siBAK1 2# sense (5’-3’), GCUUCGUGGUCGACUUCAUTT, antisense (5’-3’), AUGAAGUCGACCACGAAGCTT.

### RNA extraction and quantitative real-time PCR

Trizol reagent (Invitrogen, USA) was applied to extract total RNA according to the manufacturer’s instructions, and the NanoDrop 2000c instrument (Thermo Fisher, USA) was applied for quantifying RNA concentration. High capacity cDNA Reverse Transcription Kit (Applied Biosystems, USA) was used for the reverse transcription of 2ug total RNA. The quantitative real-time PCR (qRT‐PCR) was performed using SYBR Green Kit (Thermo Fisher, USA) and Light Cycler 480 II system (Roche, China). GAPDH were adopted for normalization with the 2^−ΔΔct^ method. Primers used were listed as follow: FDX1-F: 5-TTCAACCTGTCACCTCATCTTTG-3, FDX1-R: 5-TGCCAGATCGAGCATGTCATT-3, BAX-F: 5-CCCGAGAGGTCTTTTTCCGAG-3, BAX-R: 5-CCAGCCCATGATGGTTCTGAT-3, BAK1-F: 5-GTTTTCCGCAGCTACGTTTTT-3, BAK1-R: 5-GCAGAGGTAAGGTGACCATCTC-3, GAPDH-F: 5-AAGGTCGGAGTCAACGGATTTG-3, GAPDH-R: 5-CCATGGGTGGAATCATATTGGAA-3.

### Lipid peroxidation analysis

The experiments were conducted as previously described [21]. After treated with indicated ferroptosis inducers, cells were incubated with serum-free medium containing 5µM BODIPY-C11 for 30 min at 37 °C. After being washed three times with 1×PBS, the cells were collected and resuspended in 1×PBS for subsequent flow cytometric analysis. The oxidation of the polyunsaturated butadienyl moiety of the dye results in a shift in the fluorescence emission peak from ~ 590 nm to ~ 510 nm.

### Soluble and insoluble fraction isolation

Cells were harvested and lysed in ice-cold NP40 lysis buffer (20 mM Tris (pH 7.4), 150 mM NaCl, 1% NP40) containing protease inhibitors (EDTA-free Protease Inhibitor Cocktail, B14001, Selleck) for 30 min. Then the cell extracts were centrifuged at 14,000 rpm at 4 °C for 30 min. Finally, the supernatant and sedimentation were separated from each other. 5× SDS loading buffer was added to supernatant and the sedimentation was resuspended with 1× SDS loading buffer. Two fractions were then boiled and sent for western blotting.

### GSH assay

The relative GSH concentration in cell lysates were assessed using a GSH and GSSG Assay Kit (Beyotime, S0053) according to the manufacturer’s instructions. Simply, cell sediments were collected and treated with protein clear solution. Then the rapid freeze-thaw method was implemented. For GSSG level, lysate was pretreated with GSH clearance solution. Finally, the GSH and GSSG level was calculated by measuring the OD value at 412 nm after mixed with NAPDH solution.

### Cell growth assay

Cell growth assay was applied with the CCK-8 kit (YEASEN, 40203ES80). Briefly, cells were seeded into a 96-well plate overnight and treated as indicated, and CCK-8 reagents were added to each well, after which the plates were placed in a humidity incubator (37℃, 5%CO_2_). The absorbance measurements were carried out at 450 nm using a Thermo Scientific Spectrophotometer (1510 − 00712). Samples were prepared at least in triplicates.

### Immunofluorescence and microscopy

Cells were seeded on coverslips in 6-well plate overnight and treated as indicated. Briefly, the cells were fixed with 4% paraformaldehyde for 10 min, permeabilized in 0.25% Triton X-100 for 10 min and blotted with 3% BSA for 1 h. The cells were incubated with DLAT antibody (1:500) at 4 °C overnight. Then, the cells were washed three times with 0.1% PBS-T (PBS with 0.1% Tween-20), incubated with the appropriate secondary antibodies for 1 h at 37℃, washed and sealed with mounting medium including DAPI (Vectorlabs, H-1200). Images were captured on confocal.

For mitochondrial staining, cells were incubated with 200nM Mitotracker Red CMXRos for 30 min prior to paraformaldehyde fixation.

For cryosections immunofluorescence, the fresh tumor tissues were snap-frozen in OCT overnight and then 10µm sections were cut on a freezing microtome used for subsequent immunofluorescence analyses.

### Mice xenograft model

Male nude mice (6–8 weeks of age) were obtained from Shanghai Laboratory Animal Center and housed in the laboratory-animal research center of Zhejiang University. MHCC-97 H or QBC939 cells were resuspended with PBS, and 5 × 10^6^ of cells were subcutaneous injected into each mouse. When implanted tumors reached 100-300mm^3^, mice were randomized divided into 4 groups (n = 6) and then intraperitoneally injected with: Blank, sorafenib(10 mg/kg), elesclomol(40 mg/kg) + 0.06 mg/kg CuCl_2_, sorafenib (10 mg/kg) + elesclomol (40 mg/kg) + 0.06 mg/kg Cucl_2_ five times a week and monitored using Vernier calipers. Tumor volume (cm^3^) = 0.5 × Tumor length × Tumor width^2^. At last, the xenografts from the euthanised mice were photoed and weighed. This study was approved by the animal ethics committee of Sir Run Run Shaw Hospital, Zhejiang University School of Medicine. Animal care and experiments were conducted in compliance with Institutional Animal Care and Use Committee and NIH guidelines.

### Statistical analysis

The Student’s t-test was performed to analyze the assay results. In all experiments, p value < 0.05 was considered as a statistically significant difference (‘*’ as presented). Results were expressed as mean ± SD as indicated.

## Results

### Ferroptosis inducers enhanced copper-induced cell death in liver cancer cells

**Fig. 1:**
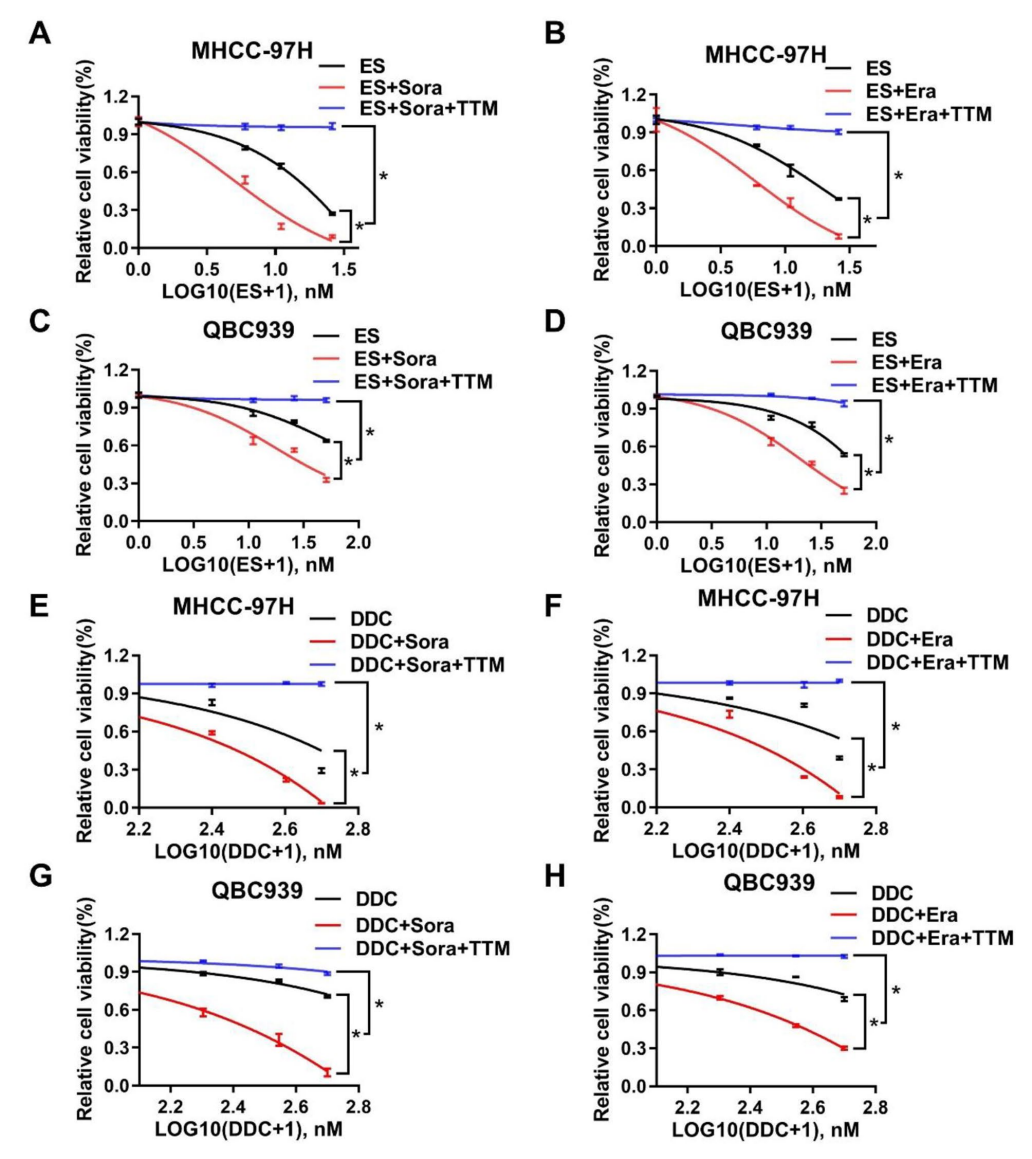
Ferroptosis inducers enhanced copper ionophores-induced cell death dependent on copper in liver cancer cells **A** and **C**. Cell viability of MHCC-97 H (**A**) or QBC939 (**C**) cells after Elesclomol (ES) 48 h treatment with DMSO, 10µM sorafenib (Sora) or 10µM Sora + 10µM TTM was measured with CCK-8 assay **B** and **D**. Cell viability of MHCC-97 H (**B**) or QBC939 (**D**) cells after Elesclomol (ES) 48 h treatment with DMSO, 10µM erastin (Era) or 10µM Era + 10µM TTM was measured with CCK-8 assay **E** and **G**. Cell viability of MHCC-97 H (**E**) or QBC939 (**G**) cells after diethyldithiocarbamate (DDC) 48 h treatment with DMSO, 10µM Sora or 10µM Sora + 10µM TTM was measured with CCK-8 assay **F** and **H**. Cell viability of MHCC-97 H (**F**) or QBC939 (**H**) cells after DDC 48 h treatment with DMSO, 10µM Era or 10µM Era + 10µM TTM was measured with CCK-8 assay. For A to G, media were supplemented with 1µM CuCl_2_

To explore the potential crosstalk between ferroptosis and cuproptosis in liver cancer cells, we firstly confirmed that sorafenib and erastin, two typical ferroptosis inducers (FINs), could dose dependently inhibit cell growth and upregulate intracellular lipid peroxidation, which indicated ferroptosis induction in HCC and ICC cells (Fig.S1A-F). Then, we found that sorafenib and erastin sensitized cell death of HCC and ICC cells induced by copper ionophore elesclomol and copper ion premixture (elesclomol-Cu) (Fig.S2A-D). Furthermore, the FINs-increased elesclomol-Cu cytotoxicity could be reversed by chelation of copper with tetrathiomolybdate (TTM) and tetraethylenepentamine (TEPA) (Fig. 1A-D and Fig.S3A-L). On the contrary, TTM did not compromise cell death triggered by sorafenib or erastin alone (Fig. S2E-L). To exclude the specificity of elesclomol, another copper ionophore diethyldithiocarbamate (DDC) was adopted. Consistent with above observations, sorafenib and erastin successfully enhanced DDC and copper premixture (DDC-Cu) triggered cell death as well, and this enhancement could be reversed by TTM too (Fig. 1E-H). In summary, FINs enhanced copper-induced cell death, which was dependent on intracellular copper in liver cancer cells.

### Ferroptosis inducers promoted cuproptosis in liver cancer cells

**Fig. 2:**
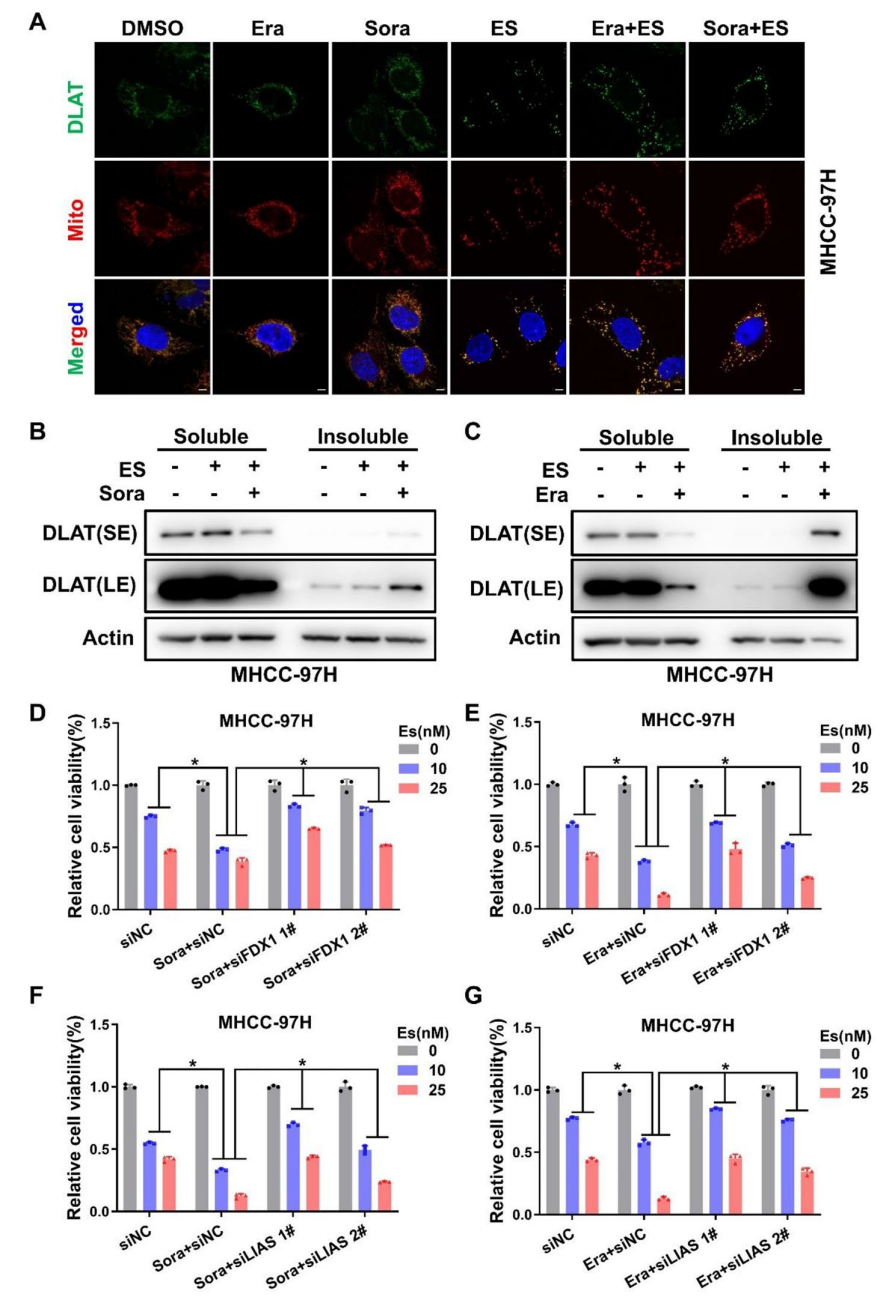
Ferroptosis inducers promoted cuproptosis in liver cancer cells **A**. MHCC-97 H cells were treated with indicated drugs for 24 h (DMSO, 10µM erastin (Era), 10µM sorafenib (Sora), 10nM elesclomol (ES), 10µM Era + 10nM ES, 10µM Sora + 10nM ES), DLAT protein aggregation was analyzed by immunofluorescence (IF) (green, DLAT; red, Mitotracker; blue, DAPI). White scale bars on full tiled images are 5μm **B** and **C**. The distribution of DLAT protein in soluble or insoluble fraction after treatment with indicated drugs for 24 h (DMSO, 10nM ES, 10µM Sora (or 10µM Era) + 10nM ES) was detected by western blotting in MHCC-97 H cells **D** and **E**. MHCC-97 H cells were transiently transfected with siFDX1, siNC was used as negative control, then the transfected cells were treated with sorafenib (Sora) or erastin (Era) and Elesclomol (ES) for another 48 h before cell viability was measured with CCK-8 assay **F** and **G**. MHCC-97 H cells were transiently transfected with siLIAS, siNC was used as negative control, then the transfected cells were treated with sorafenib (Sora) or erastin (Era) and Elesclomol (ES) for another 48 h before cell viability was measured with CCK-8 assay. For A to G, media were supplemented with 1µM CuCl_2_

Next, we wanted to determine whether FINs-sensitizing effect was due to enhanced cuproptosis. As a newly defined regulated cell death, cuproptosis is characterized by aggregation of lipoylated protein such as dihydrolipoamide S-acetyltransferase (DLAT) [20]. Thus, immunofluorescence (IF) experiments were carried out to visualize the distribution of DLAT in liver cancer cells. Indeed, elesclomol-Cu led to the formation of DLAT puncta, which meant the aggregation of DLAT. In addition, co-treatment of sorafenib or erastin together with elesclomol-Cu significantly augmented DLAT aggregation in liver cancer cells compared to elesclomol-Cu alone (Fig. 2A and Fig.S4A). As previously reported, mutant huntingtin protein (mHTT) has been proven to undergo a process that is prone to aggregate with each other and decrease its solubility, which could be detected by the isolation of insoluble fraction [22]. As expected, the soluble DLAT protein level decreased and the insoluble DLAT increased under elevated elesclomol-Cu incubation (Fig.S4B), and co-incubated sorafenib or erastin dramatically enhanced elesclomol-Cu augmented insoluble DLAT in liver cancer cells (Fig. 2B-C and Fig.S4C). Previously, those proteins involved in protein lipoylation, such as ferredoxin 1 (FDX1) and lipoyl synthase (LIAS), have been shown to be upstream regulators of cuproptosis through upregulating protein lipoylation [20]. Furthermore, FDX1 has also been identified as a target of elesclomol which could reduce Cu^2+^ to Cu^1+^ [23, 24]. Either LIAS or FDX1 knockdown could significantly reverse the copper induced cytotoxicity [20]. Actually, we did confirm that knockdown of LIAS or FDX1 could reverse elesclomol-Cu induced DLAT aggregation (Fig.S5A-D). In line with these findings, knockdown of LIAS or FDX1 could also reverse ferroptosis inducers promoted elesclomol-Cu induced cell death (Fig. 2D-G and Fig.S5E-H). Previous reports suggested that elesclomol could promote cell death through the induction of apoptosis [25]. Therefore, we conducted further exploration to determine whether apoptosis is involved in this sorafenib and erastin-sensitized effect. Our investigation showed that neither pan-caspase inhibitors (Z-VAD-FMK and Boc-D-FMK) treatment nor BAX and BAK1(the main apoptosis effectors) knockdown rescued ferroptosis inducers enhanced cell death (Fig. S6A-J). Taken together, these results indicated that FINs promoted copper induced cuproptosis in liver cancer cells.

### Ferroptosis inducers promoted cuproptosis through depleting intracellular GSH

**Fig. 3:**
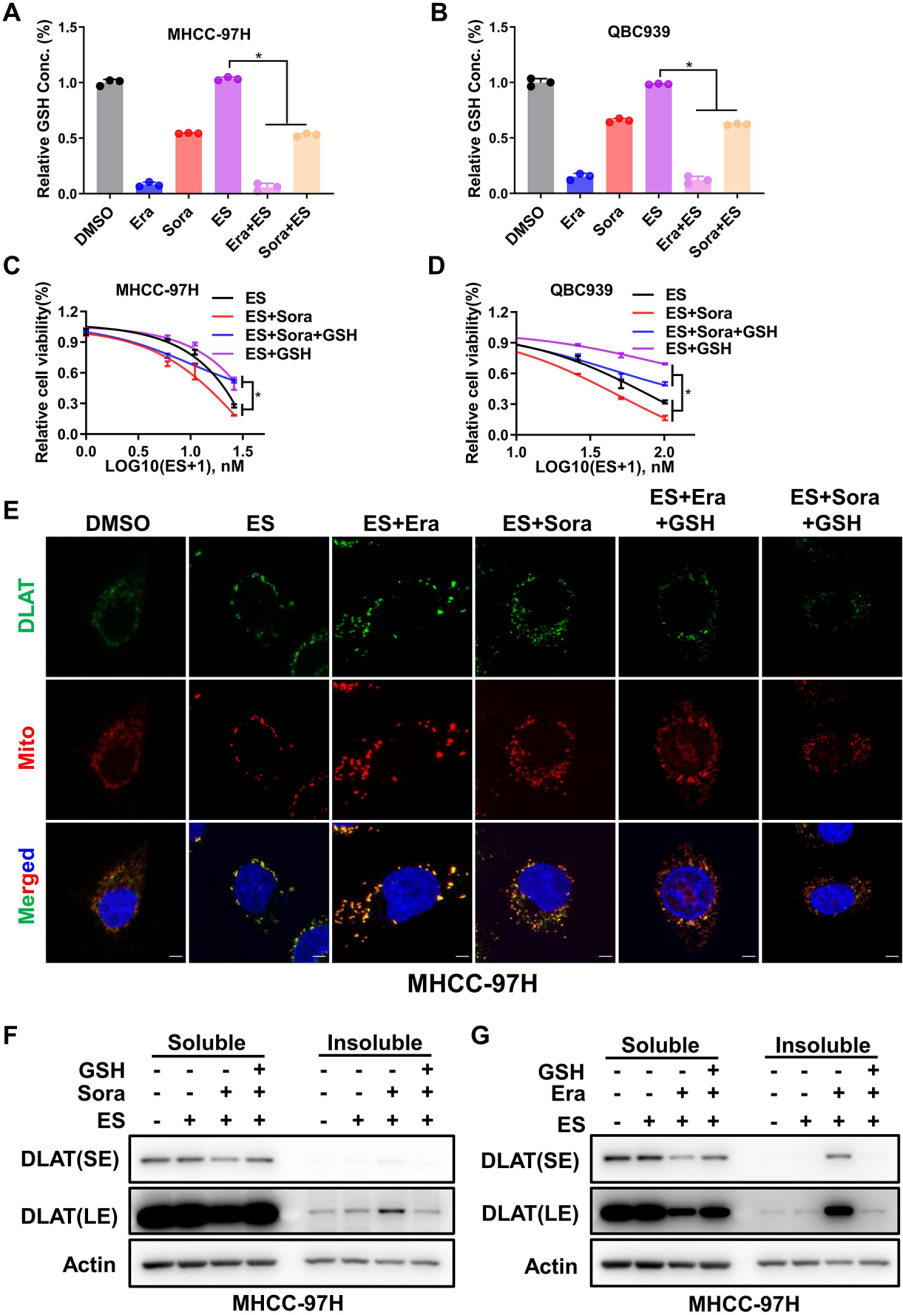
Ferroptosis inducers promoted cuproptosis through depleting intracellular GSH **A** and **B**. Relative GSH level in MHCC-97 H (**A**) or QBC939 (**B**) cells after treatment with DMSO, 10µM erastin (Era), 10µM sorafenib (Sora), 10nM elesclomol (ES), 10µM Era + 10nM ES or 10µM Sora + 10nM ES. C and D. Cell viability of MHCC-97 H (**C**) or QBC939 (D) cells after ES 48 h treatment with DMSO, 10µM Sora, 10mM GSH or 10µM Sora + 10mM GSH was measured with CCK-8 assay **E**. MHCC-97 H cells were treated with indicated drugs for 24 h (DMSO, 10nM ES, 10µM Era + 10nM ES, 10µM Sora + 10nM ES, 10µM Era + 10nM ES + 10mM GSH, 10µM Sora + 10nM ES + 10mM GSH), DLAT protein aggregation was analyzed by immunofluorescence (green, DLAT; red, Mitotracker; blue, DAPI). White scale bars on full tiled images are 5μm **F** and **G**. The effect of GSH (10mM) on elesclomol (ES 10nM) and Sora (10µM) or Era (10µM)-induced DLAT protein aggregation was analyzed by western blotting in MHCC-97 H cells. For **A** to **G**, media were supplemented with 1µM CuCl_2_

Previous studies have found that ferroptosis inducers like sorafenib and erastin could downregulate intracellular GSH synthesis through inhibiting system Xc^−^ to promote ferroptosis [26]. In addition, GSH has been proven to be an alternative copper chelator [20]. Therefore, we asked whether GSH bridged the crosstalk between ferroptosis and cuproptosis. Firstly, we confirmed that sorafenib or erastin could reduce intracellular GSH content in liver cancer cells treated with FINs alone or FINs plus elesclomol-Cu (Fig. 3A-B). Moreover, supplementation of GSH reversed the sensitizing effect of sorafenib or erastin on copper-induced cytotoxicity (Fig. 3C-D and Fig.S7A-B). In contrast, increased DLAT aggregates upon FINs treatment were eliminated remarkably after GSH replenishing (Fig. 3E-G and Fig.S7C-E). These results suggested that ferroptosis inducers promoted cuproptosis through depleting intracellular GSH in liver cancer cells.

### Ferroptosis inducers promoted protein lipoylation through suppressing mitochondria dependent FDX1 protein degradation

**Fig. 4:**
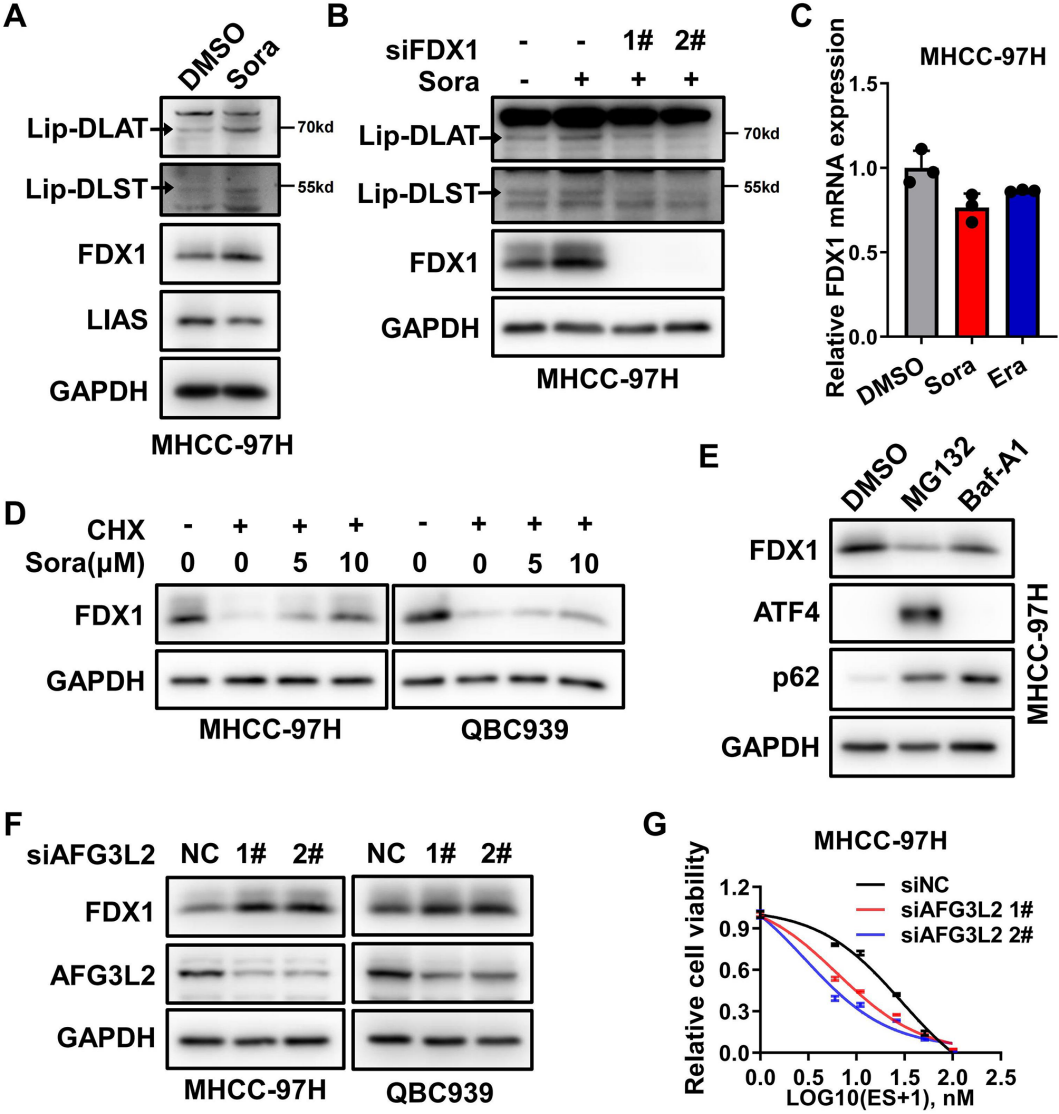
Ferroptosis inducers promoted protein lipoylation through suppressing FDX1 protein mitochondria proteases dependent degradation **A**. DLAT and DLST protein lipoylation (lip-DLAT and lip-DLST), FDX1 and LIAS protein level in MHCC-97 H cells treated with 10µM sorafenib (Sora) for 24 h were blotted with indicated antibodies, GAPDH was used as the loading control **B**. lip-DLAT, lip-DLST, FDX1 protein level in MHCC-97 H cells with FDX1 knocking down and Sora (10µM) treatment for 24 h were blotted with indicated antibodies **C**. FDX1 mRNA expression in MHCC-97 H cells treated with Sora or Era 24 h was determined by real-time RT-PCR. **D**. The effect of sorafenib on FDX1 protein stability in MHCC-97 H (left) or QBC939 (right) cells with Cycloheximide (CHX, 100 µg/ml) and indicated concentration of Sora 12 h treatment was analyzed by immunoblotting **E**. FDX1 protein level in MHCC-97 H cells with DMSO, 10µM MG132 or 100nM Baf-A1 treatment for 12 h was analyzed by immunoblotting. ATF4 and p62 were used as positive controls of MG132 and Baf-A1 respectively **F**. The effect of AFG3L2 knockdown on FDX1 expression was analyzed by western blotting after 48 h of siRNA transfection in MHCC-97 H (left) or QBC939 (right) cells **G**. Cell viability of MHCC-97 H cells with Elesclomol-Cu after AFG3L2 knockdown in MHCC-97 H cells was measured with CCK-8 assay

Besides the intracellular copper chelator, protein lipoylated status has been shown to be essential for cuproptosis [20]. We next further explored whether FINs could alter the lipoylation level of proteins such as DLAT or DLST. Interestingly, either sorafenib or erastin treatment resulted in the elevated level of lip-DLAT or lip-DLST (Fig. 4A and Fig.S8A). As previous reports and our above findings, LIAS and FDX1 are the two critical upstream proteins responsible for protein lipoylation. Interestingly, we found that FDX1, but not LIAS, was increased after sorafenib or erastin treatment (Fig. 4A and Fig.S8A). Furthermore, knockdown of FDX1 could rescue sorafenib or erastin-induced upregulation of lip-DLAT and lip-DLST (Fig. 4B and Fig.S8B). These results suggested that sorafenib or erastin could augment protein lipoylation through upregulating FDX1.

Subsequently, we analyzed how FDX1 was upregulated under sorafenib or erastin treatment. Our results showed that sorafenib or erastin could not increase the mRNA level of FDX1, but significantly stabilized FDX1 protein (Fig. 4C-D and Fig.S8C). However, inhibition of two major protein degradation pathways, proteasome and lysosome, failed to raise the protein level of FDX1(Fig. 4E), indicating that it might be other uncommon protein control system involved in the regulation of FDX1 protein level by ferroptosis inducers. Accumulating evidence suggests the importance of mitochondrial proteases in modulating biochemical activities that are critical for mitochondrial function such as protein quality control [27]. FDX1 has been shown to present in the mitochondrial matrix, so we next focused on the three mitochondrial matrix-related proteases, AFG3L2 complex, LONP1 and CLPP complex [27]. While all three mitochondrial matrix-related proteases might participate in the turnover of FDX1, AFG3L2 complex seems to be the dominant one (Fig. 4F and Fig.S8D-E). Consistently, AFG3L2 knockdown indeed sensitized liver cancer cell to copper-induced cell death (Fig. 4G). Taken together, our data supported that FINs could upregulate protein lipoylation level through suppressing FDX1 protein degradation mediated by mitochondrial matrix-related proteases, which contributed to ferroptosis inducers enhanced cuproptosis.

### Ferroptosis inducers promoted cuproptosis in liver cancer in vivo

**Fig. 5:**
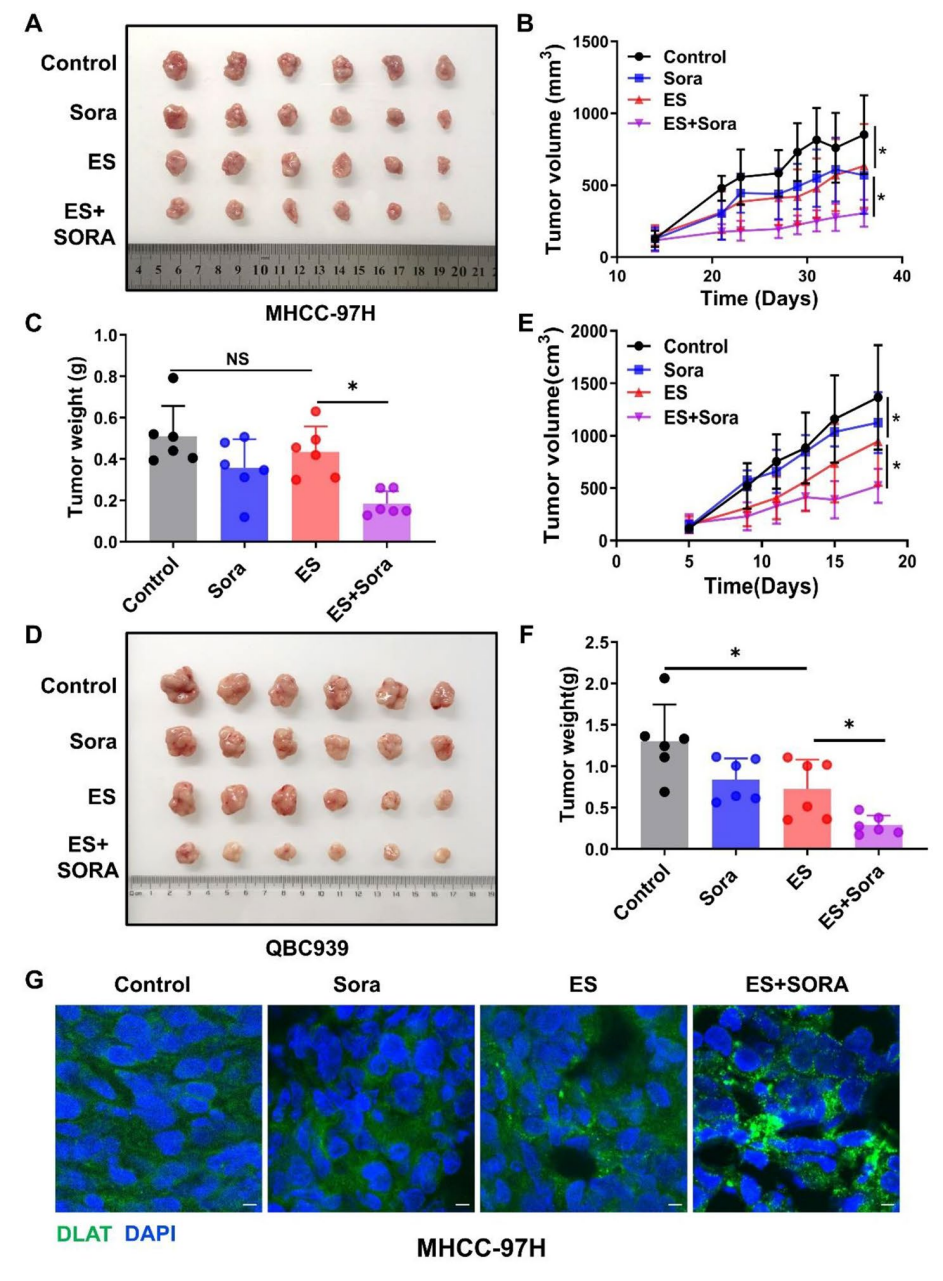
Ferroptosis inducers promoted cuproptosis in liver cancer in vivo **A**-**F**. Nude mice xenograft model (n = 6 per group) was generated by subcutaneous inoculation of MHCC-97 H (**A**-**C**) or QBC939 (**D**-**F**) cells. Mice were then treated with indicated drugs. Tumor pictures (**A** and **D**), tumor growth curve (**B** and **E**) and tumor weight (**C** and **F**) were summarized and shown respectively **G**. Frozen tissue sections from each group of MHCC-97 H xenograft model were labeled with DLAT (green) and DAPI (blue). White scale bars on full tiled images are 5μm

Based on the above findings, we further explored the synergy effect of elesclomol-Cu and sorafenib in vivo. Consistent with in vitro results, the nude mice xenograft model showed that elesclomol-Cu combined with sorafenib significantly retarded in vivo HCC and ICC cell growth compared to either single drug administration (Fig. 5A-F). Furthermore, IF staining exhibited that DLAT aggregates was more pronounced in combined group compared to either single drug treatment group (Fig. 5G and Fig.S9A). Taken together, the xenograft studies confirmed that ferroptosis inducer sorafenib promoted cuproptosis in liver cancer in vivo.

## Discussion

**Fig. 6:**
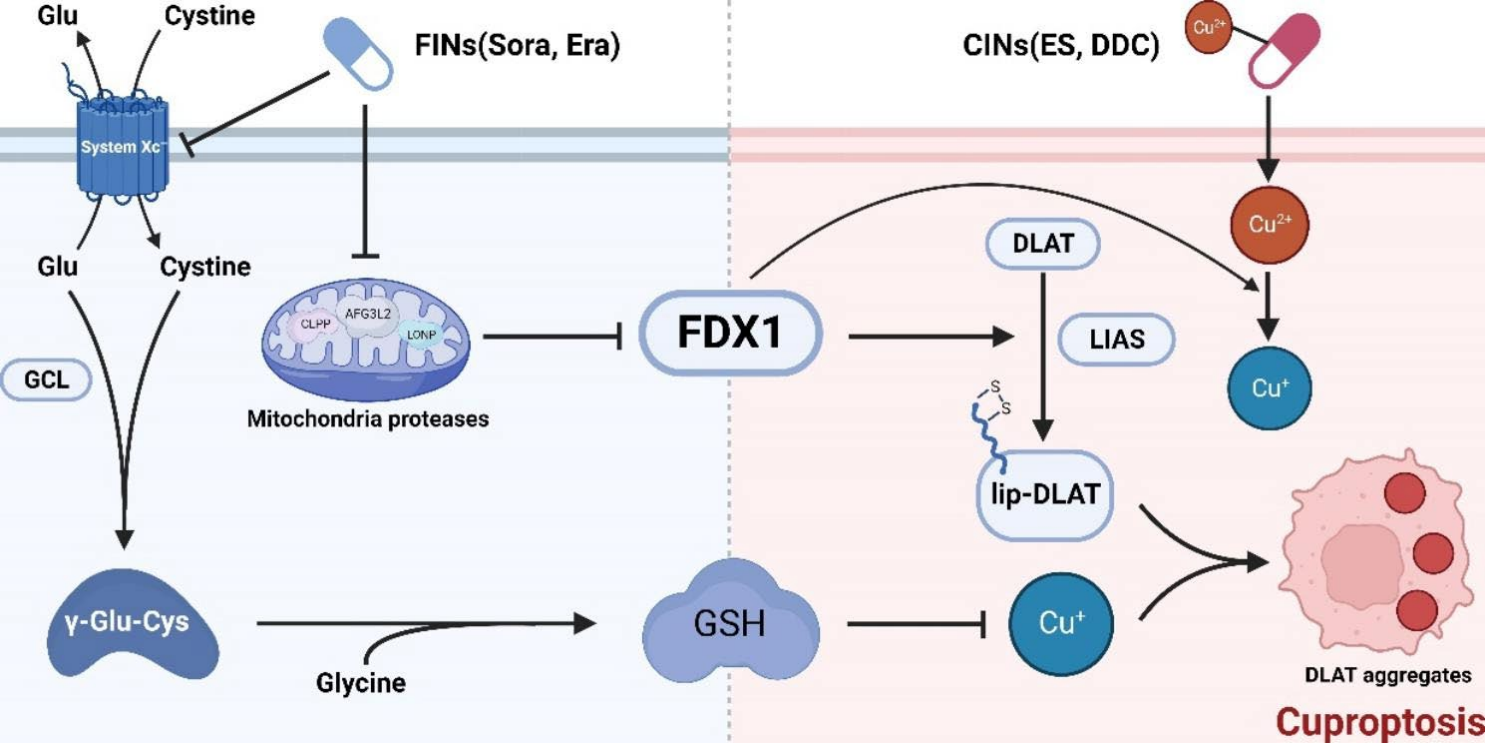
Schematic diagram of ferroptosis inducers enhanced cuproptosis induced by copper ionophores in primary liver cancer Ferroptosis inducers (FINs) sorafenib (Sora) and erastin (Era) promoted copper ionophores (CINs)-induced cuproptosis through stabilizing FDX1 protein and depleting intracellular GSH level. Mechanically, FINs stabilized FDX1 protein by suppressing mitochondrial proteases (AFG3L2 etc.) mediated FDX1 protein turnover. The stabilized FDX1 enhanced the protein lipoylation process and promoted the transfer of reduced copper ion, Cu^1+^. In addition, FINs could also inhibit the import of cystine through suppressing the catalytic subunit (SLC7A11) of system Xc^−^, resulting in the decrease of GSH level, which could elevate the concentration of liable copper ion. Overall, these factors together augmented the aggregation of lipoylated proteins, and thus promoted cuproptosis in liver cancer cells. The diagram was created from BioRender.com

Liver cancer is a fatal cancer with high incidence worldwide. The occurrence and progression of liver cancer is such a complex process with a series of genetic and epigenetic alterations involved that effective therapies are still limited clinically [28–31]. It is of urgent need to design new treatment strategies for liver cancer. Ferroptosis and cuproptosis are the two newly identified modes of regulated cell death form dependent on metal ion. Here we found that ferroptosis inducers sorafenib and erastin could upregulate protein lipoylation via suppressing mitochondrial matrix-related proteases mediated FDX1 protein degradation, and suppress intracellular GSH synthesis through inhibiting cystine importing, which eventually enhanced copper-induced cuproptosis (Fig. 6).

Copper and iron are essential mineral nutrients acting as cofactor or signaling metal that play critical roles in multiple cellular process such as antioxidant defense, mitochondrial respiration, angiogenesis and tumor progression including initiation, growth and metastasis [5, 32, 33]. Since excessive level of intracellular copper and iron are cytotoxic, the intracellular level of copper and iron are fine-tuned by active homeostatic metabolism network. Thus, targeting metal metabolism homeostasis to induce cell death has been successfully translated to clinical cancer therapy, especially for the well-established iron-dependent ferroptosis, such as the administration of sorafenib in liver cancer [7]. Moreover, the copper-induced cell death also exhibits the clinical potential, such as administration of elesclomol in melanoma [4]. The discovery and definition of cuproptosis recently has significantly advanced the researches on targeting copper metabolism in cancer. Cuproptosis-based nanomaterial has been applied in bladder cancer and it has been shown to enhance the efficacy of immunotherapy by combining with anti-programmed cell death protein ligand-1 antibody (αPD-L1) [34, 35]. Moreover, the prognostic model constructed by cuproptosis-related genes has demonstrated excellent predictive capacity for patient outcomes in various cancers, such as liver cancer, breast cancer and lower-grade gliomas [36–38]. Additionally, the level of cuproptosis-related genes may impact the sensitivity of patients to immunotherapy, radiotherapy, and chemotherapy [38].

The crosstalk between different regulated cell death (RCD) such as necroptosis, pyroptosis, ferroptosis, and cuproptosis has been gradually noticed [33]. However, the crosstalk between cuproptosis and ferroptosis is still not clarified. Liu et al. constructed the ferroptosis potential index(FPI) and cuproptosis potential index(CPI), and suggested that ferroptosis and cuproptosis may both involved in the anti-hepatocellular carcinoma effect of curcumin [39]. Previous studies found that excessive copper ion delivered by copper ionophore disulfiram disrupted mitochondrial homoeostasis and caused oxidative stress. In addition, disulfiram promoted intracellular iron accumulation and lipid peroxidation which finally resulted in ferroptosis [40]. In the current study, we unveiled that ferroptosis inducers could enhance copper induced cuproptosis in liver cancer cells, presenting a novel strategy for the treatment of liver cancer.

On one side, GSH is an alternative chelator of copper that controls the concentration of liable copper ion pool [20]. On the other side, GSH acts as the substrate for GPX4 to reduce lipid peroxidation. Thus, it is well known that sorafenib and erastin could promote ferroptosis mainly by suppressing the catalytic subunit (SLC7A11) of system Xc^−^, which imports cystine for the synthesis of intracellular GSH [26]. Meanwhile, as a novel form of regulated cell death distinct from ferroptosis and other regulated cell deaths, the major hallmark of cuproptosis is the aggregation of lipoylated proteins using copper ion as a core. And copper chelators such as TTM and GSH could rescue copper-induced cuproptosis [20]. Therefore, GSH might be the key component to mediate the crosstalk of ferroptosis and cuproptosis. As a result, ferroptosis inducers did decrease intracellular GSH level, and replenishing of GSH weakened DLAT aggregation and subsequent cuproptosis enhanced by sorafenib and erastin (Fig. 3). Taken together, either inhibition of GSH synthesis by ferroptosis inducers or chelating of intracellular GSH by copper would magnify copper/iron induced cell death.

Besides labile copper ion pool, protein lipoylation is also implicated in cuproptosis. It is mainly regulated by lipoylation upstream regulators FDX1 and LIAS, lipoylated targeted protein DLAT, DLST and so on. Indeed, we confirmed that knockdown of FDX1 or LIAS not only reduced elesclomol-Cu induced DLAT aggregation, but also alleviated ferroptosis inducers promoted cuproptosis (Fig. 2). Furthermore, we found ferroptosis inducers could upregulate protein lipoylation level through stabilizing FDX1 protein. And mitochondrial matrix proteases, but not the regular protein degradation system such as proteasomal and lysosomal degradation, was further identified as the novel pathway for FDX1 protein turnover (Fig. 4). Hence, managing the protein lipoylation process might be another feasible strategy to target cuproptosis in cancer. However, how FINs retard mitochondrial proteases-mediated FDX1 protein degradation needed further investigations.

## Conclusions

Over all, we demonstrated a crosstalk between ferroptosis and cuproptosis, in which ferroptosis inducers enhanced cuproptosis through protein lipoylation activation and GSH synthesis suppression. Our results proposed that combination of sorafenib and elesclomol-Cu, two FDA approval drugs, to co-target ferroptosis and cuproptosis could be a potential strategy for liver cancer treatment.

## Electronic supplementary material

Below is the link to the electronic supplementary material.

Supplementary Material 1

## Data Availability

Not applicable.
