# Supplementary material for: Ferroptosis inducers enhanced cuproptosis induced by copper ionophores in primary liver cancer
Source: J Exp Clin Cancer Res. 2023 Jun 6;42:142. doi: 10.1186/s13046-023-02720-2 (PMC10242978; doi:10.1186/s13046-023-02720-2)
Supplement: Supplementary file 1 — Supplementary Material 1 [file 13046_2023_2720_MOESM1_ESM.docx]

**Supplemental Figures and Legends**

**
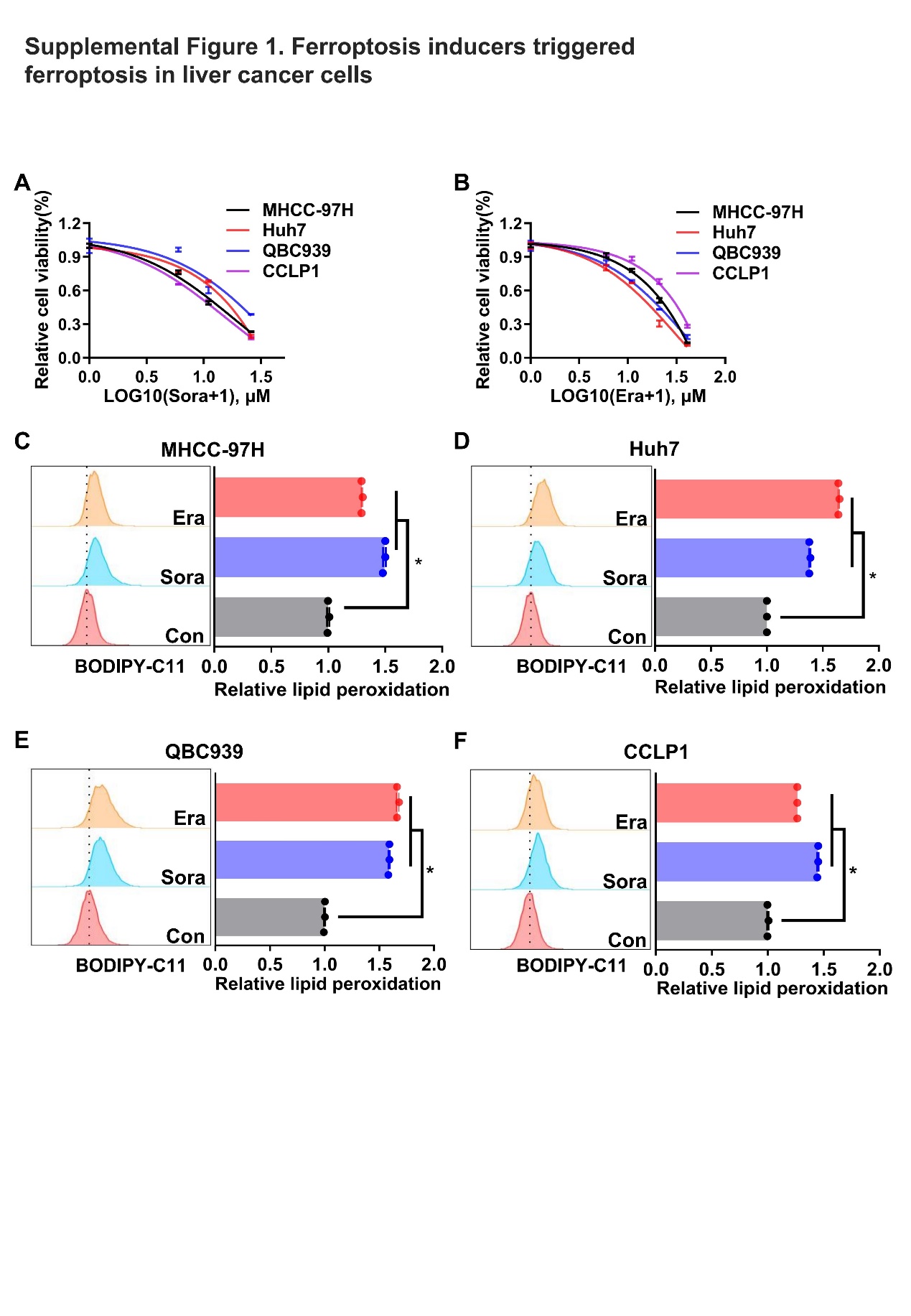
**

**Supplemental Figure 1. Ferroptosis inducers triggered ferroptosis in liver cancer cells**

A. Cell viability of MHCC-97H, QBC939, Huh7 and CCLP1 cells with indicated dose of sorafenib (Sora) treated for 48h were determined by CCK-8 assay.

B. Cell viability of MHCC-97H, QBC939, Huh7 and CCLP1 cells with indicated dose of erastin (Era) treated for 48h were determined by CCK-8 assay.

C-F. Lipid peroxides of MHCC-97H, QBC939, Huh7, CCLP1 cells after treated with DMSO, 10μM sorafenib (Sora) or 10μM erastin (Era) for 48h, were detected with BODIPY-C11.

**
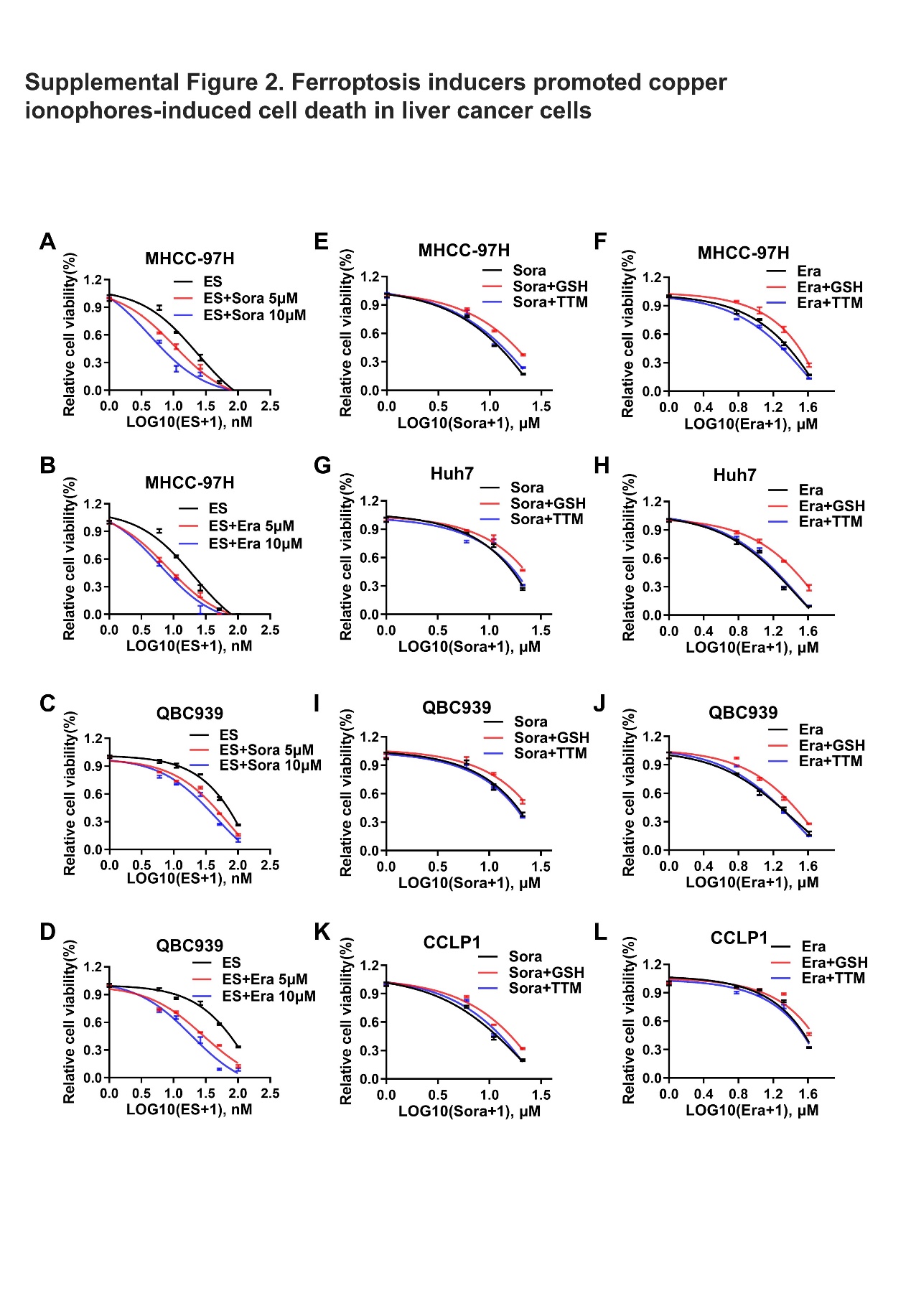
**

**Supplemental Figure 2. Ferroptosis inducers promoted copper ionophores-induced cell death in liver cancer cells**

A and B. Cell viability of MHCC-97H cells after Elesclomol (ES, 48h) treatment with sorafenib (Sora, 0, 5, 10μM) or erastin (Era, 0, 5, 10μM) was measured with CCK-8 assay.

C and D. Cell viability of QBC939 cells after Elesclomol (ES, 48h) treatment with Sora (0, 5, 10μM) or Era (0, 5, 10μM) was measured with CCK-8 assay.

E-L. Cell viability of MHCC-97H, Huh7, QBC939, CCLP1 cells after Sora or Era treatment with DMSO, 10mM GSH or 10μM TTM together were measured with CCK-8 assay.

For A to D, media were supplemented with 1μM CuCl_2_.


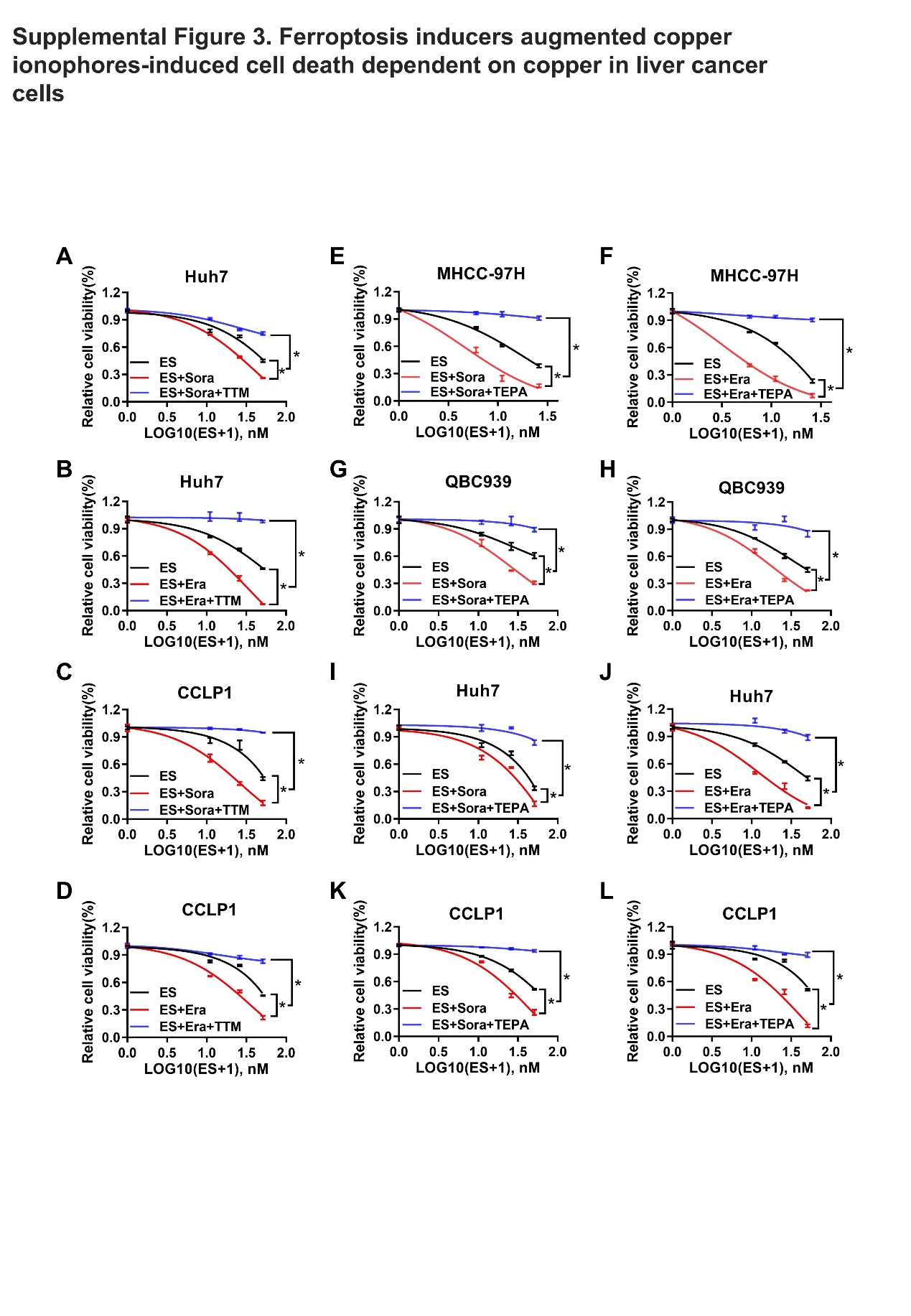


**Supplemental Figure 3. Ferroptosis inducers augmented copper ionophores-induced cell death dependent on copper in liver cancer cells**

A and C. Cell viability of Huh7 or CCLP1 cells after Elesclomol (ES) 48h treatment, together with DMSO, 10μM sorafenib (Sora) or 10μM Sora + 10μM TTM was measured with CCK-8 assay.

B and D. Cell viability of Huh7 or CCLP1 cells after Elesclomol (ES) 48h treatment, together with DMSO, 10μM erastin (Era) or 10μM Era + 10μM TTM was measured with CCK-8 assay.

E-L. Cell viability of MHCC-97H(E-F), QBC939(G-H), Huh7(I-J), CCLP1(K-L) after Elesclomol (ES) 48h treatment, together with DMSO, 10μM Sora (or 10μM Era) or 10μM Sora (or 10μM Era) + 5mM TEPA were measured with CCK-8 assay.

For A to L, media were supplemented with 1μM CuCl_2_.


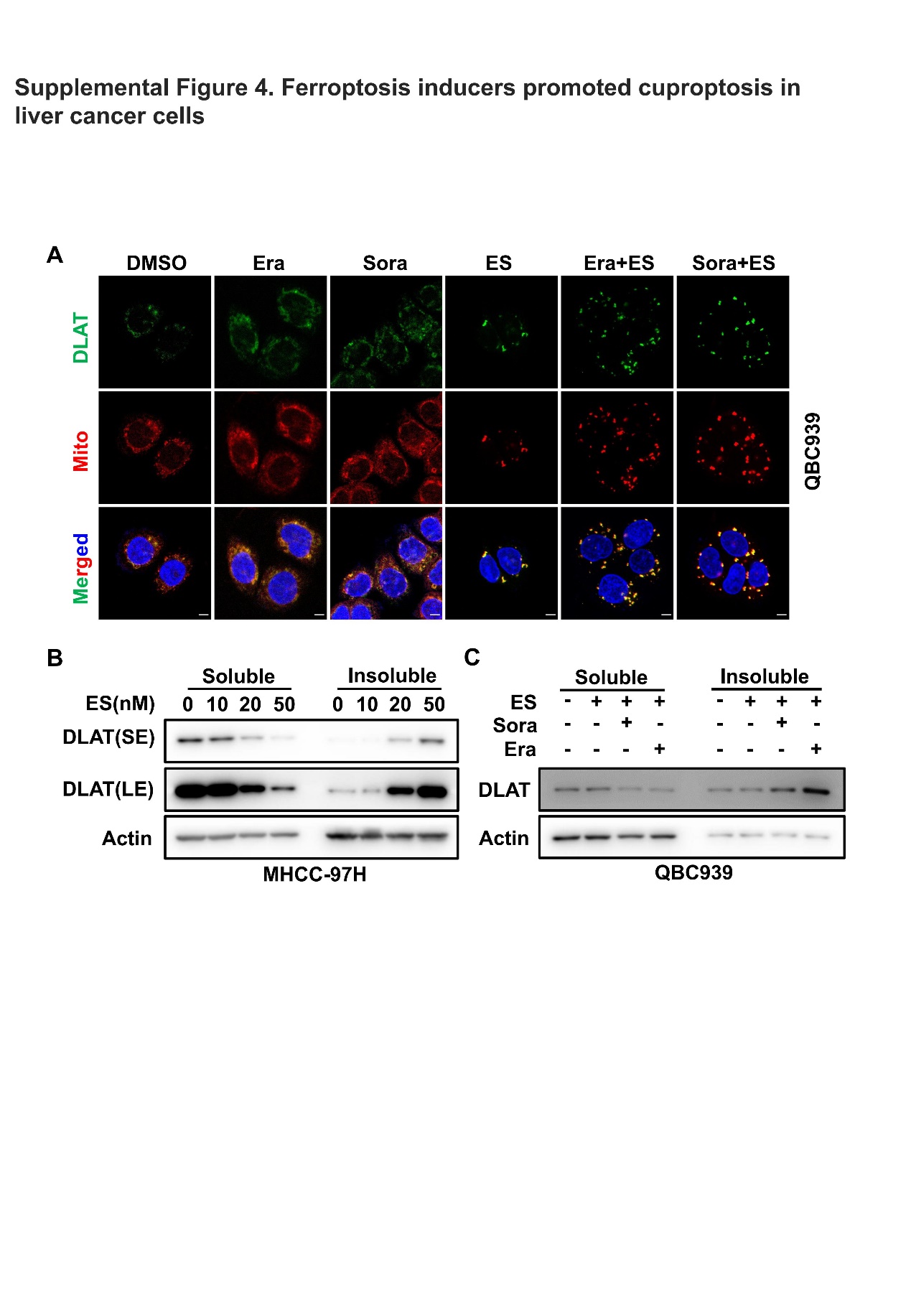


**Supplemental Figure 4. Ferroptosis inducers promoted cuproptosis in liver cancer cells**

A, QBC939 cells were treated with indicated drugs for 24h (DMSO, 10μM erastin(Era), 10μM sorafenib(Sora), 10nM elesclomol(ES), 10μM Era + 10nM ES, 10μM Sora +10nM ES), DLAT protein aggregation was analyzed by immunofluorescence imagine (green, DLAT; red, Mitotracker; blue, DAPI). White scale bars on full tiled images are 5μm.

B, The distribution of DLAT protein in soluble or insoluble fraction in MHCC-97H cells after treatment with indicated drugs for 24h (DMSO, 10nM ES, 20nM ES, 50nM ES) was detected by western blotting.

C, The distribution of DLAT protein in soluble or insoluble fraction in QBC939 cells after treatment with indicated drugs for 24h (DMSO, 10nM ES, 10nM ES+10μM sora, 10nM ES+10μM Era) was detected by western blotting.


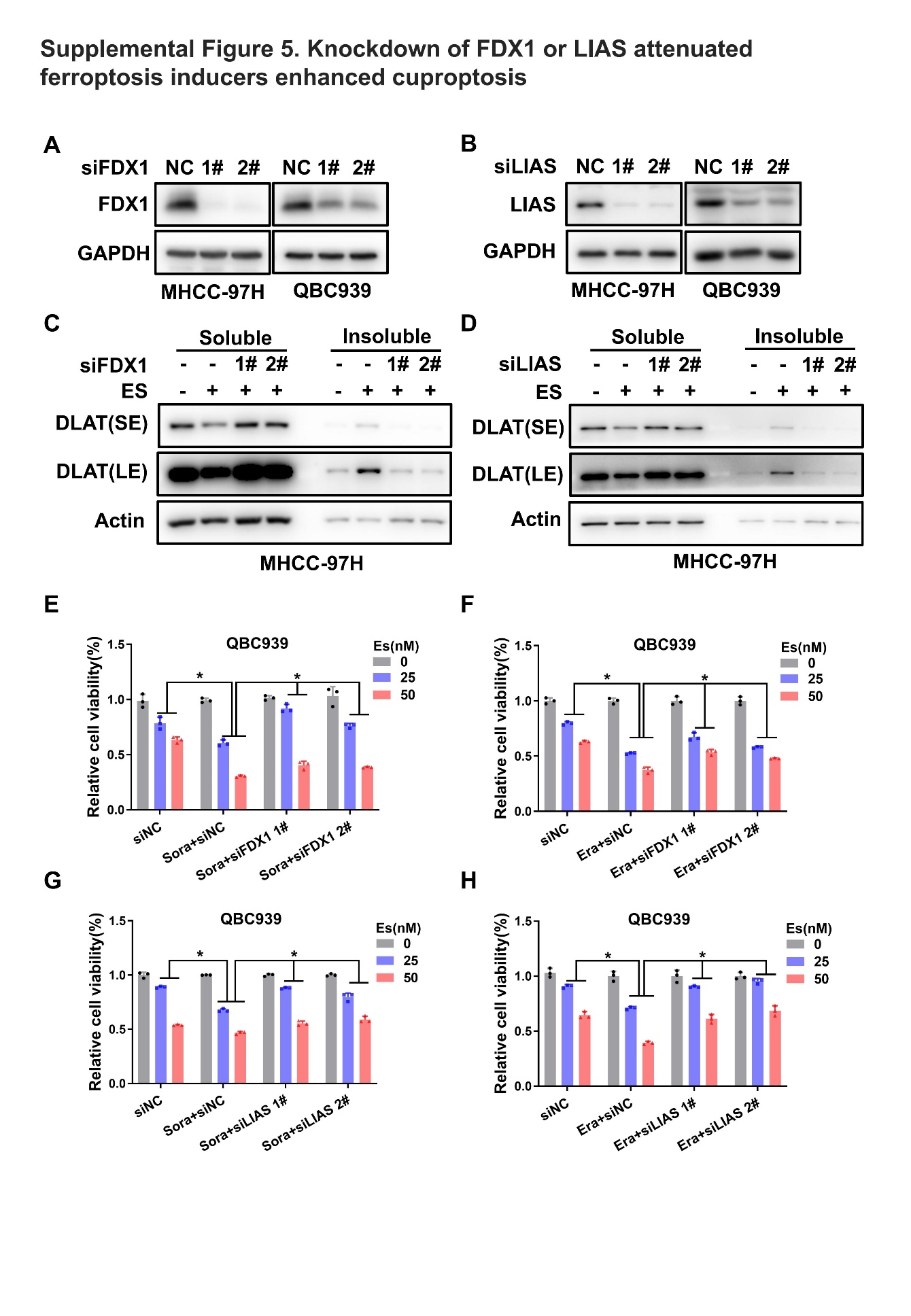


**Supplemental Figure 5. Knockdown of FDX1 or LIAS attenuated ferroptosis inducers enhanced cuproptosis**

A and B. The knockdown efficiency of FDX1 by siFDX1 (A) or LIAS by siLIAS (B) in MHCC-97H (left) or QBC939 (right) cells was measured with western blotting.

C and D. The effect of LIAS or FDX1 knockdown on the distribution of DLAT protein in soluble or insoluble fraction under 20nM ES treatment was detected by Western blotting.

E and F. After knockdown of FDX1 in QBC939 cells with siFDX1, cells were treated with Sora (E) or Era (F), together with Elesclomol (ES) for 48h, cell viability was measured with CCK-8 assay.

G and H. After knockdown of LIAS in QBC939 cells with siLIAS, cells were treated with Sora (E) or Era (F), together with Elesclomol (ES) for 48h, cell viability was measured with CCK-8 assay.

For C to H, media were supplemented with 1μM CuCl_2_.


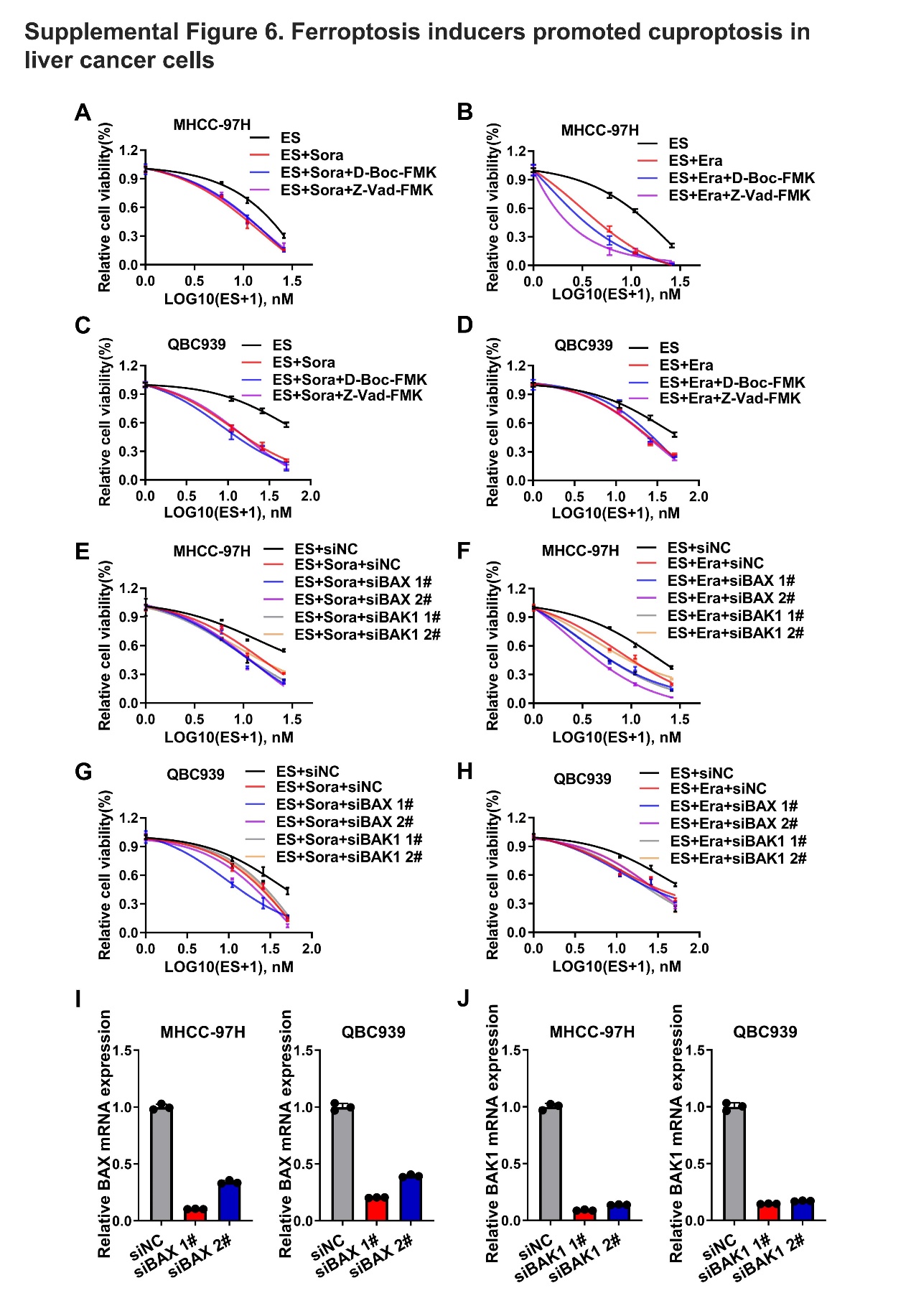


**Supplemental Figure 6. Ferroptosis inducers promoted cuproptosis in liver cancer cells**

A and C. Cell viability of MHCC-97H or QBC939 cells after Elesclomol (ES) 48h treatment, together with DMSO, 10μM sorafenib (Sora), 10μM Sora+20μM D-Boc-FMK or 10μM Sora+50μM Z-Vad-FMK was measured with CCK-8 assay.

B and D. Cell viability of MHCC-97H or QBC939 cells after Elesclomol (ES) 48h treatment, together with DMSO, 10μM erastin (Era), 10μM Era+20μM D-Boc-FMK or 10μM Era+50μM Z-Vad-FMK was measured with CCK-8 assay.

E and G. After BAX or BAK1 knockdown with siRNA transient transfection, cell viability of MHCC-97H or QBC939 cells treated together with Elesclomol (ES) and 10μM sorafenib (Sora) for 48h, was measured with CCK-8 assay.

F and H. After BAX or BAK1 knockdown with siRNA transient transfection, cell viability of MHCC-97H or QBC939 cells treated together with Elesclomol (ES) and 10μM erastin (Era) for 48h, was measured with CCK-8 assay.

I and J. The knockdown efficiency of the BAX and BAK1 was validated by qRT-PCR in MHCC-97H and QBC939 cells.

For A to H, media were supplemented with 1μM CuCl_2_.

**
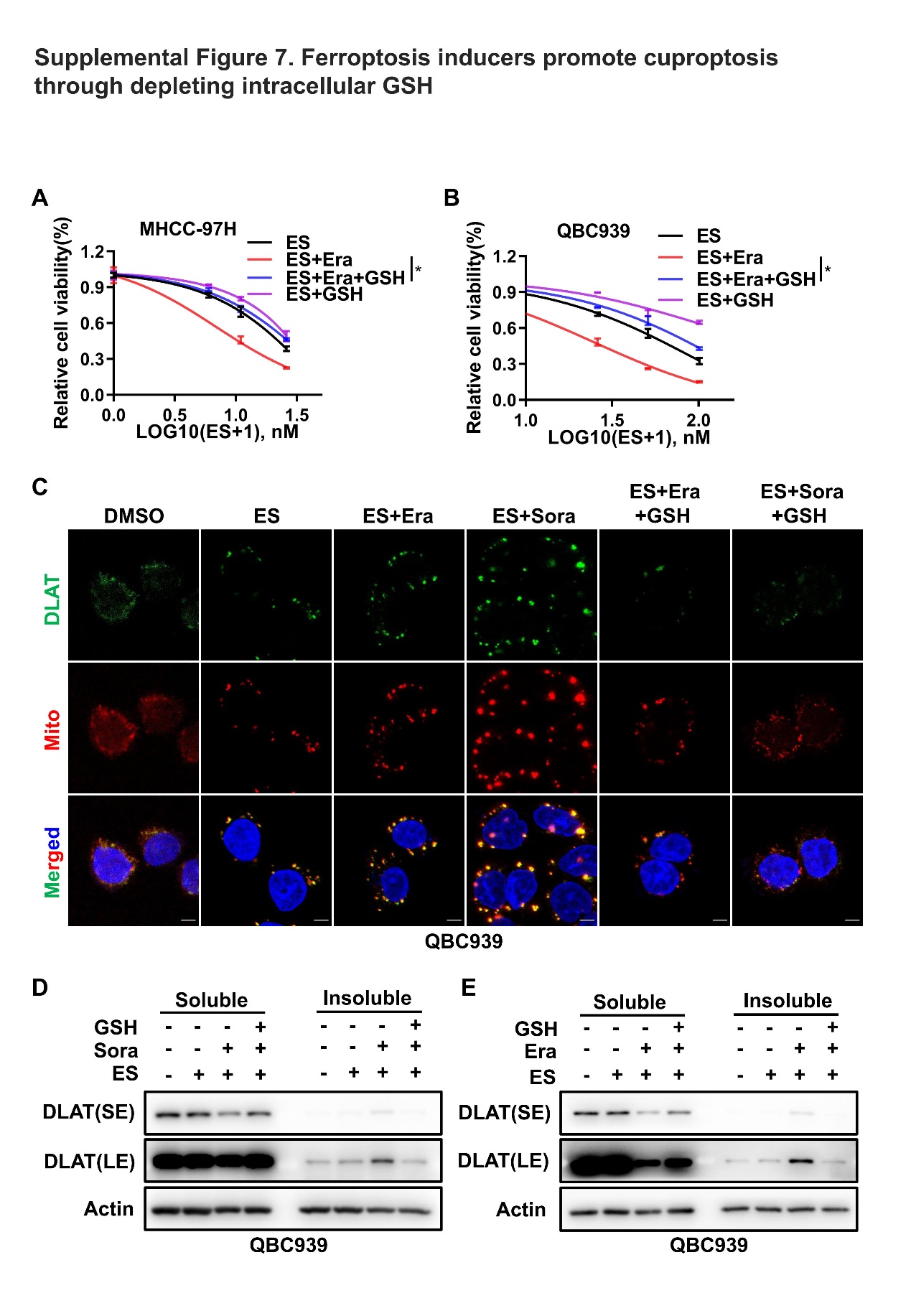
**

**Supplemental Figure 7. Ferroptosis inducers promoted cuproptosis through depleting intracellular GSH**

A and B. Cell viability of MHCC-97H or QBC939 cells after Elesclomol (48h) treatment with DMSO, 10μM Erastin or 10μM Erastin+ 10mM GSH or 10mM GSH was measured with CCK-8 assay.

C. QBC939 cells treated with indicated drugs for 24h (DMSO, 10nM elesclomol, 10μM erastin+ 10nM elesclomol, 10μM sorafenib+10nM elesclomol, 10μM erastin+ 10nM elesclomol+ 10mM GSH, 10μM sorafenib+10nM elesclomol+ 10mM GSH), protein aggregation was analyzed by immunofluorescence imagine (green, DLAT; red, Mitotracker; blue, DAPI). White scale bars on full tiled images are 5μm.

D and E. The effect of GSH (10mM) on 10μM Sora or 10μM Era plus 10nM ES-induced DLAT protein aggregation was analyzed by western blotting in QBC939 cell.

For A to E, media were supplemented with 1μM CuCl_2_.


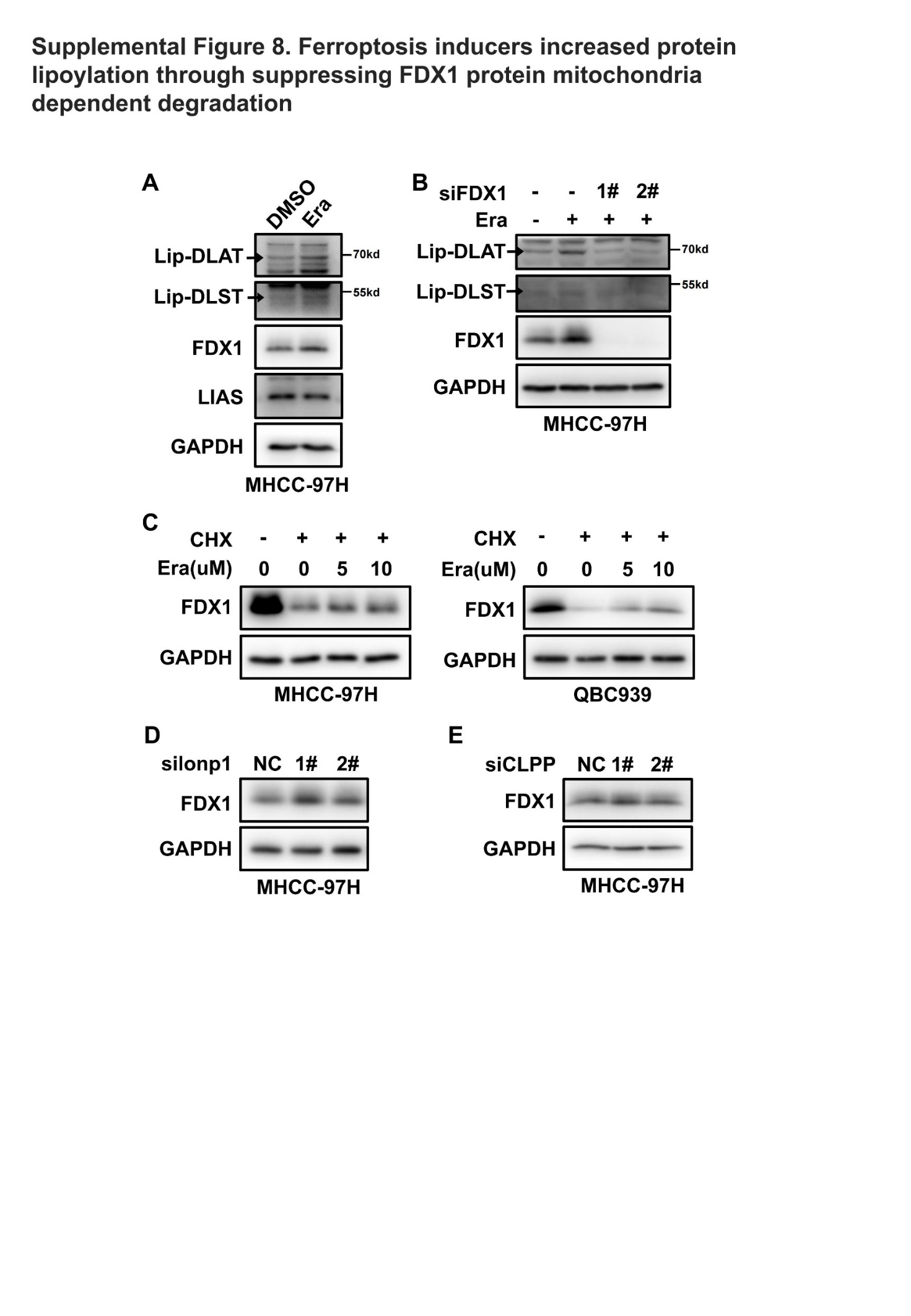


**Supplemental Figure 8. Ferroptosis inducers increased protein lipoylation through suppressing FDX1 protein mitochondria dependent degradation**

A. Immunoblot of lipoylated proteins (lip-DLAT and lip-DLST), FDX1, LIAS and GAPDH from MHCC-97H cells treated with 10μM erastin for 24h.

B. Immunoblot of lip-DLAT, lip-DLST, FDX1 from extracts of MHCC-97H cells with indicated treatment: siNC+DMSO, siNC+10μM Era, siFDX1 1#+10μM Era, siFDX1 2#+10μM Era.

C. The effect of erastin on FDX1 protein stability in MHCC-97H (left) or QBC939 (right) cells with Cycloheximide (CHX, 100 μg/ml) and indicated concentration of erastin 12h treatment was analyzed by immunoblotting.

D and E. The effect of lonp1 knockdown or CLPP knockdown on FDX1 expression was analyzed by western blotting after 48h of transfection in MHCC-97H cells.


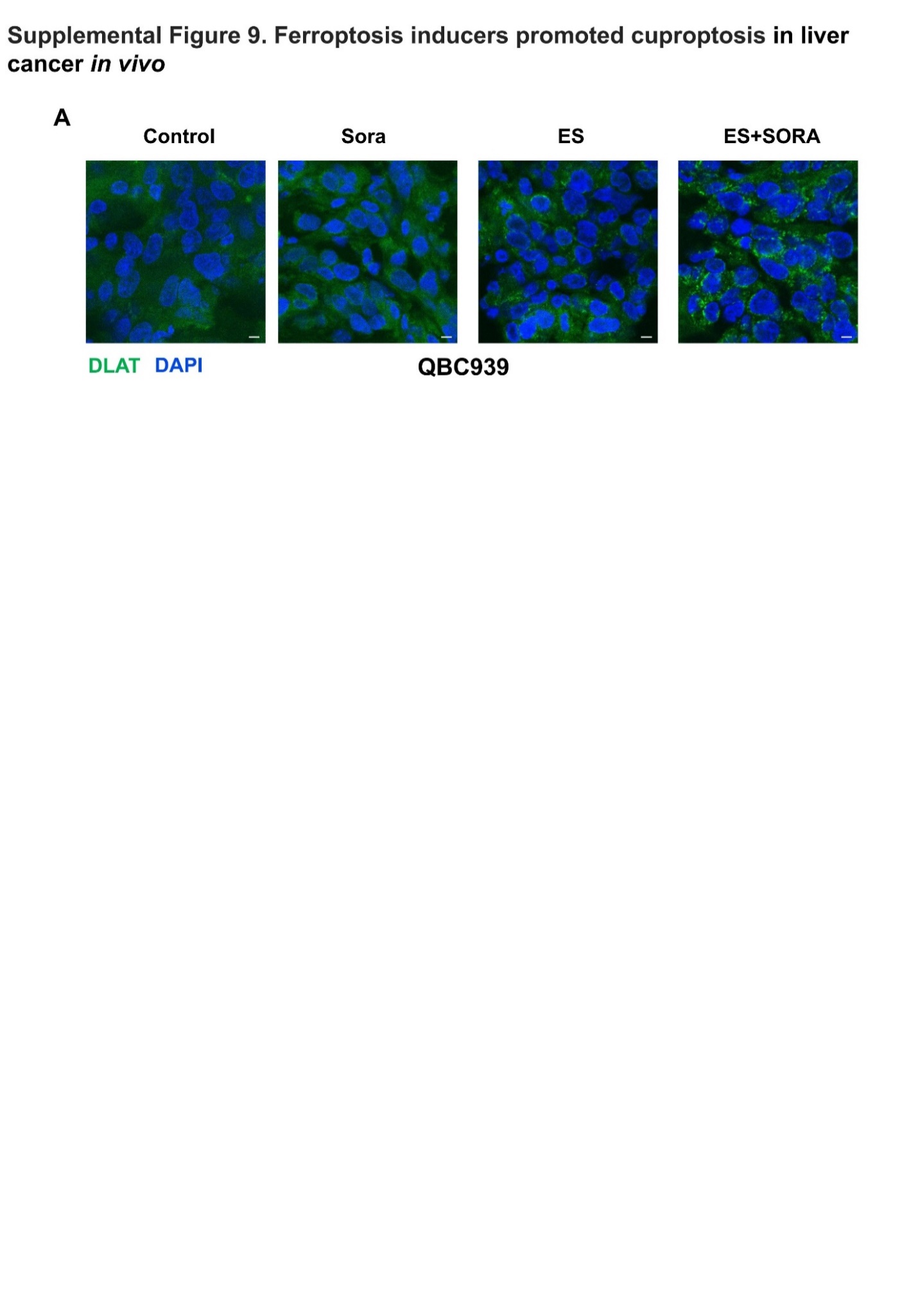


**Supplemental Figure 9. Ferroptosis inducers promoted cuproptosis in liver cancer *in vivo***

A. Frozen tissue sections from each group from QBC939 xenograft model were labeled with DLAT (green) and DAPI (blue). White scale bars on full tiled images are 5μm.
